# Environmental factors influencing Prevention and Control of Schistosomiasis Infection in Mwea, Kirinyaga County Kenya: A cross sectional study

**DOI:** 10.24248/eahrj.v5i1.656

**Published:** 2021-06-11

**Authors:** Judy Mwai, Jarim Oduor Omogi, Mohamed H. Abdi

**Affiliations:** a Kenya Medical Research Institute; b Jomo Kenyatta University of Agriculture and Technology

## Abstract

**Background:**

Schistosomiasis remains a major public health problem in Kenya. Environmental factors are critical in creating a medium for growth and spread of schistosomiasis vectors. The study investigated the environmental factors influencing prevention and control of schistosomiasis infection in Mwea West Sub County, Kirinyaga County-Kenya.

**Methods:**

A multi stage sampling was used to identify four hundred and sixty-five (465) household. Analytical descriptive cross-sectional design that utilised quantitative data collection method was used. Data was collected using a pretested structured questionnaire and analysed using Chi square tests or Fisher's exact tests where applicable.

**Results:**

Study results indicated a significant association *p<.001* between household level of education, members being affected by floods during the rainy season and schistosomiasis infection. The result further indicates level of significance (*p<0.047*) in the association between sources of water in a household and schistosomiasis infection. No level of significance was posted between having a temporary water body in the area *p (=.072)* and schistosomiasis infection. In addition, there was no significant association between proximity to the nearest water source, *p=.074* and proximity to the nearest health facility *p=0.356* with schistosomiasis infection.

**Conclusions:**

The study recommends carefully designing safe water sources in order to match the goal of effectively controlling and reversing the trends of schistosomiasis infections. The community should be made aware of the risk factors of schistosomiasis including water utilised in the household's alongside raising health seeking behaviours for diagnosis and treatment of schistosomiasis as a way of reducing the spread of infection.

## BACKGROUND

Schistosomiasis is among the Neglected Tropical Diseases (NTDs) targeted for control by the World Health Organization.^1^ According to WHO, 218 million individuals suffer from schistosomiasis globally while 700 million are at risk in 76 endemic regions. According to the report, Kenya was ranked among the 10 highest burden countries in the African region with almost 12 million people said to be in need of Preventive Chemotherapy (PC) while treatment coverage approximated to be zero.^2^

The burden of schistosomiasis cannot be wished away with literature suggesting that the disease accounts for an estimated 1.9 million Disability Adjusted Life Years (DALYs) annually with 90% of the burden currently concentrated in Africa. Though school age children have been the focus for both treatment and epidemiologic evaluation because of their high risk of infection, schistosomiasis infection and morbidity risk extends to preschool-aged children, women of reproductive age, and other high-risk groups (e.g., car washers, fishermen, and rice farmers). This suggest that treatment of additional at-risk groups is required in order to achieve comprehensive morbidity control.^3^

The two (2) main species of schistosomiasis in Kenya are S. mansoni and S. haematobium with approximately 2.5 million people feared to be at risk of infection.^4^

Transmission of schistosomes is said to be through human contact patterns to infested water, the presence of competent intermediate snail hosts, availability of suitable snail hosts habitats, and freshwater environment contamination with stool/urine containing eggs. The distribution of the disease mainly depends on the presence of *Bulinus* spp. and *Biomphalaria* spp. as intermediate host snails for *S. haematobium* and *S. mansoni*, respectively.^5^

In Kenya, schistosomiasis occurs mostly in western, coast, and selected foci in central part of the country.^6^ Preventive anthelminthic chemotherapy usually through Mass Drug Administration (MDA) programs is the preferred and prioritised first line strategy recommended by the World Health Organization (WHO) to overcome the burden and morbidity inflicted by these infections. Additionally, interventions targeting improvement of access to Water, Sanitation and Hygiene (WASH) are encouraged as long-term a-nd sustainable control measure.^7^

Schistosomiasis has been known to contribute significantly to lower social economic conditions in areas where it is endemic and causes a great deal of disability thus reducing the work performance among the infected individuals.^8^

Previous studies including the one conducted by Ministry of Public Health and Sanitation and Ministry of Education in conjunction with Japan International Cooperation Agency (JICA), initiated a schistosomiasis control program based on mass treatment in Mwea West Sub County. A total of 43,928 school age children from 86 schools were de-wormed by trained school teachers. The prevalence of the parasitic infections in the 5 cohort schools was 38 % for *S. mansoni* before treatment. There was an overall parasitic re-infection rate of 16 % for *S. mansoni*, 6 months after treatment. The trend of re-infection continued after treatment to 22 % in the second year, 31 % in the third year and 17 % in the fourth year.^6^

A proper understanding of disease prevalence and the effects of the environment will not only provide a useful tool for proper planning of effective control programmes but also form a basis of exploring other potential adverse health related effects instigated by schistosomiasis. This study aimed at investigating environmental factors influencing the prevention and control of schistosomiasis in Mwea West Sub-County Kirinyaga County Kenya.

## METHODS

### Study Area

The study was conducted in Kangai and Thiba locations in Mwea West Sub County. Mwea Sub County is one of the 5 sub counties of Kirinyaga county. Kirinyaga County covers an area of 1,478.1 Square Kilometres. The County lies between 1158 M and 5380 M above sea level in the South and at the Peak of Mount Kenya respectively. The 2019 Kenya Population and Housing Census (KPHC) put the population of the County at 610,411 and Mwea sub County at 125,962^9^. The mean annual rainfall is approximated to be 1200 and 1600 mm per year. The Sub County is home to Mwea irrigation scheme where a number of water canals crisscross the area supplying irrigation water to the farms and villages respectively. The main socio-economic activities include rice and horticultural farming.^10^ In a publication by Kenya National Bureau of Statistics (KNBS) and Society for International Development (SID),^11^ only 34% of residents use improved source of water with only 35% of residents having homes with cement floors. According to the Kirinyaga County Integrated Development Plan 2018–2022^10^, the county has 202 health facilities comprising of 109 public health institutions, 39 Mission/NGO facilities and 54 private clinics.

The largest Mission Health facility is the Mwea Mission hospital, in Mwea Sub County. There are 3 level four hospital facilities located in Kirinyaga Central, Gichugu and Mwea Constituencies. The County has 348 Early childhood development (ECD) centres, 326 primary schools, 143 secondary schools and 29 tertiary institutions.

### Study Design

The study employed an analytical descriptive cross-sectional design adopting quantitative data approach to investigate environmental factors influencing the prevention and control of schistosomiasis in Mwea West sub-County Kirinyaga County.

### Sample Size Determination

The minimum sample size was computed using the formula by.^12^ The current prevalence is unavailable, thus an assumed prevalence of 50% was used in the computation of the minimum sample size required for the study with a 5% margin of error and a design effect of 1.2%. The following equations provides the determination of the sample size.


(1) $$n=\frac{Z^{2}p(1-p)}{d^{2}}\times DEFF$$

Where;

n= is the minimum calculated sample size for populations greater than 10,000

Z= Standard errors from mean corresponding to the 95% confidence level is 1.96

P= the target prevalence, assumed 50%, p=0.5

d= the level of statistical significance (allowable error / precision) of 5%, d=0.05

DEFF= Design Effect

Thus,


(2) $$n=\frac{1.96^{2}\times 0.5(1-0.5)}{0.05^{2}}\times 1.2$$

(3) $$n=\frac{3.8416\times 0.5\times 0.5}{0.0025}\times 1.2=\frac{1.15248}{0.0025}=460$$

Allowing for a non-response rate of 1% gave a final adjusted sample size of 465 as below


(4) $$n=460\times \frac{101}{100}=464.6∼465$$

### Data Collection

The main instrument used for quantitative data collection was a structured questionnaire. The questionnaire was translated into Kiswahili. The investigators recruited research assistants who were conversant with Mwea Sub County and also who are proficient in the Kikuyu language.

A total of 465 household heads were enrolled in the study by use of simple random sampling technique. Having carried out probability proportional to size and identified the households that acted as the sampling frame, the former method was used in identifying the specific households visited using 10 as the n^th^ in skipping 1 household to the other. A random walk was utilised in identifying the first household. The study largely used Last Birthday method for within household selection due to its accuracy.^13^ Prior to data collection that took a total of 7 days, 10 research assistants were recruited and trained for 2 days and a pretest conducted on the second day. In the evening of the same day, all gaps identified on the tool were corrected accordingly before embarking on data collection. The training focused on the objective of the research, understanding of the questionnaire, modalities of data collection and how to collect data. During the training, both English, Swahili and kikuyu languages were used for better understanding of the questionnaire. The main issues - captured in the questionnaire included socio-demographic characteristics and environmental factors. The 10 field assistants were employed to assist in administering the questionnaire under close supervision of the principal investigator and biostatistician. The questionnaire took an average of 30 to 35 minutes to administer

### Data Management and Analysis

Quantitative data collected was entered into Microsoft Excel and Microsoft Access 2019 Office Application Software and statistical analysis done after data validation. Data from the questionnaires were then fed into the IBM Software, the Statistical Package for Social Scientists (SPSS) version 23.^14^ Descriptive statistics including mean, or median, frequencies and proportions were appropriately generated. Chi square test was used to test associations between variables.

### Ethical Considerations

This study was reviewed and approved by the KEMRI Ethical Review Committee (SSC/ERC protocol No. 2061). The study used questionnaires uniquely coded with results of each questionnaire being kept in strict confidence. Participating in the study was voluntary and participants could withdraw at any point. Participants were assured of confidentiality and that no names will appear in any report. Written informed consent was obtained from participants before embarking on the study.

## RESULTS

### Socio-Demographic Characteristics of the Respondents

Out of 465 participants, females formed the majority 297(63.9%) with 292(63%) of the participants being married while 94(20.2%) of participants were either widowed, single or divorced. A majority of the participants 463(99.6%) were Christians with Muslims and others constituting only 2(0.4%). 66.7% of the total participants had attained primary level education while the rest had either no formal education, secondary education or post-secondary. Further analysis on occupation of the participants revealed that 368(79.1%) were farmers while less than 1% were unemployed as shown in Table 1.

**TABLE 1:** Socio-Demographic Characteristic of Respondents

Variable	Response	Frequency (Percentage (%))
**Gender**	Male	168 (36.1%)
	Female	297 (63.9%)
**Marital Status**	Married	371 (63.0%)
	Single	45 (9.7%)
	Divorced	23 (4.9%)
	Widow	20 (4.3%)
	Widower	6 (1.3%)
**Religion**	Christian	463 (99.6%)
	Muslim	1 (0.2%)
	Other	1 (0.2%)
**Level of Education**	No Formal Education	34 (7.3%)
	Primary Education	311 (66.9%)
	Secondary Education	107 (23.0%)
	Post-Secondary	11 (2.4%)
**Occupation**	Public Servant	4 (0.9%)
	Farmer	368 (79.1%)
	Business	32 (6.9%)
	Informal Employment	59 (12.7%)
	Not Employed	2 (0.4%)
**Age group (years)**	17–26 years	160 (34.4%)
	27–36 years	132 (28.4%)
	37–46 years	60 (12.9%)
	46–56 years	49 (10.5%)
	57–66 years	40 (8.6%)
	67–76 years	18 (3.9%)
	77–86 years	6 (1.3%)

### Environmental Factors associated with Schistosomiasis Infection

Less than half of the participants 212(45.6%) stated that they lived in mud houses, 187(40.2%) lived in wooden houses while majority 333(71.6%) of the respondents’ houses had earthed floor. In addition, almost all 447(96.1%) participants used pit latrines in their homestead and more than half 267(57.4%) of the participants used canal as their main source of water. Almost all participants stated that the proximity to the nearest water source and a health facility from their homestead was between 1 and 5 kilometres (km) away while 358(77%) had temporary water bodies in their locality as shown in Table 2.

**TABLE 2:** Distribution of Responses on Environmental Factors

Questions	Response	Frequency (n=465)	Percentage
**Type of house structure?**	Concrete Blocks	25	5.4
	Stone Building	41	8.8
	Mud House	212	45.6
	Wooden	187	40.2
**Type of flooring in the house?**	Cement/tiles	67	14.4
	Wooden planks	61	13.1
	Earth/Sand	333	71.6
	NA	4	0.9
**Type of toilet in the homestead?**	Toilet	5	1.1
	Pit latrine	447	96.1
	None	10	2.2
	Other	3	0.6
**Source of water for drinking?**	Piped/tap water	13	2.8
	Rainwater	23	4.9
	Stream/river	148	31.8
	Canal	267	57.4
	Others	14	3
**Distance to the water source**	Less than km	1	0.2
	1km–5km	460	98.9
	6km–10km	2	0.4
	11km–15km	2	0.4
**Presence of swamps/rivers in**	Yes	358	77
	No	107	23
**Distance to health facility**	1km–5km	461	99.1
	6km–10km	3	0.6
	11km–15km	1	0.2

### Associations between Demographics, Environmental factors and Schistosomiasis Infection

Table 3 summarises the results for the association between *S mansoni* infection and household demographic variables and environmental.

**TABLE 3:** Distribution of Environmental Factors and Having Suffered From Schistosomiasis Infection

Description	Having suffered from *S mansoni* infection		n (%)	*Statistical Significance*
	Yes n(%)	No n(%)		
**Gender of the respondents**				
Male	158(94.0)	7(6.0)	168(100)	p=.060
Female	268(90.2)	29(9.8)	297(100)	
**Level of Education of the respondents**				
Primary	112(36.01)	199(63.99)	311(100)	<.001
Secondary	48(44.86)	59(55.14)	107(100)	
Post-Secondary	5(45.45)	6(54.55)	11(100)	
Not Educated	2(5.88)	32(94.12)	34(100)	
**Member of household infected by schistosomiasis during rainy season**				
Yes	181(93.8)	12(6.2)	193(100)	<.001
No	68(53.1)	60(46.9)	128(100)	
**Sources of Water**				
Pipe/Tap Water	6(46)	7(54)	13(100)	p<.047
Rain Water	19(83)	4(17)	23(100)	
Stream/River	103(70)	45(30)	148(100)	
Canal	94(73)	73(27)	267(100)	
Other	8(62)	3(38)	13(100)	
**Proximity of homestead to the nearest water source**				
Less than 1km	0(0)	1(100)	1(100)	p=.074
1km–5km	326(71)1	34(29)	460(100)	
6km–8km	2(100)	0(0)	2(100)	
11km–15km	2(100)	0(0)	2(100)	
**Presence of temporary water bodies such as swamps and rivers in the area**				
Yes	261(73)	97(27)	358(100)	p=.072
No	69(64)	38(36)	107(100)	
**Proximity of homestead to the nearest health facility**				
1km–5km	326(71)	135(29)	461(100)	p=.356
6km–10km	2(100)	0(0)	2(100)	
11km–15km	1(100)	0(0)	1(100)	

Generally, female participants had the highest infection than male participants, though not statistically significant (*p=.060*). Households with the highest primary education level were the most infected compared to those who had secondary level with a strong significance (*p =.001*). The results also show that the households were much likely 436(93.8%) to be infected with *S mansoni* during rainy seasons with a strong significance *(p=.0001)*. Source of water remain an important factor when it comes to infections with households fetching their water from canals 340(73.1%) and rivers 326(70.1%) likely to be infected (*p=0.047*) compared to the households getting piped water and rainwater. Though a majority 330(71%) of the households indicated that they travel between 1 and 5 km to their nearest water source and that they have temporary water bodies like swamps and rivers 340(73.1%), no statistical significance was deduced. There was no significance either between having suffered from *S mansoni* and proximity of homestead to the nearest health facility.

## DISCUSSIONS

Most of the NTDs including Schistosomiasis control interventions are largely focused on preventive chemotherapy which is implemented through school based programs.^8^

According to our findings, males were more affected compared to female. The findings are similar to a study in Migori Kenya.^15^ In another study done in Sudan, though not statistically significant, boys were found more affected than girls.^16^

According to WHO, there is need for integrated approach to overcome the global impact of NTDs through 5 interventions: innovative and intensified disease management; preventive chemotherapy; vector ecology and management; veterinary public-health services; and the provision of safe water, sanitation and hygiene.^2^ The percentage of households accessing improved source of water for drinking is much less than the national figures that stands at 88.2% in urban and 59.1% in rural areas^11^ indicating a significant problem in accessing this important basic need.

In our study, male were more affected than female and this concurs with a study in Northern Ghana^17^ and Zambia^18^ that found high level of infection among male than female. Female did not show statistical significant association with schistosomiasis infection in our study. There has been inconsistent association of sex with schistosomiasis infection. The association between gender and schistosomiasis infection varies in different communities where some studies have reported association with female gender.^19^ It is now clear that men and women have different water contact behaviour relating to activities among them swimming, fishing, farming and even doing laundry.

In our findings, the prevalence of schistosomiasis infection reduced with increase in education. The respondents with primary education level were more likely to be infected in comparison to those who had reached secondary or post-secondary level of education with a statistical significance of*p=.001.* The education level may affect the behaviour and attitude of the individuals. For instance, those with low education may spend more of their time in water hence exposing themselves. In addition, they are likely not to use protective gears while at their working sites. Our study is similar to a study by^19^ which revealed that those educated had significantly better knowledge on the signs and symptoms, transmission (snail) and prevention of schistosomiasis when compared to their counterparts. It is also similar to a cross sectional community based field study conducted in Nigeria where the risk of infection was higher in those with primary school education.^20^ In contrast, a previous study from Uganda found no significant association between educational level and the level of knowledge on schistosomiasis.^21^ As far as education plays an important role in people's perceptions and practices of controlling schistosomiasis,^22^ previous studies from Africa and Asia showed that the odds of having lower knowledge about schistosomiasis were significantly higher in the respondents who had primary education level or below.^23,24^

Study results indicate that there is a significant association between households being affected by floods during the rainy season and household members having schistosomiasis infections. The findings are similar to a study done in Eastern China where there was high number of human infections that occurred during flooding of the lake.^25^ It is key to note that; (i) floods and associated sediment input create and sustain suitable snail habitat, (ii) floods are a major source of introduction and re-introduction of snails, (iii) floods lead to the admixture of different parasite lineages to which snail populations may not be well adapted, and (iv) Inundation following heavy rains helps sustain suitable habitat for free-swimming parasite larvae.

While it is clear that sanitation breaks the transmission cycle of many diseases, a number of factors influence the degree to which disease protection is afforded i.e seasonality; which has general impacts on the transmission of schistosomiasis diseases. The season can also have impacts on the sanitation facilities themselves with heavy rains causing pit latrines and sewerage systems to flood and become inoperable and possibly contaminate the environment. The current study finding concur with systematic review that found people with safe water and adequate sanitation having significantly lower odds of a *Schistosoma* infection.^26^

The present results reveal significance association between sources of water in a household and schistosomiasis infection. This concurs with a cross sectional study in South Africa where learners who went to an open source of water for their domestic needs had a 64.2% infection rate. In the same study, the prevalence increased with decreasing distance to the water body.^27^ The frequency and type of water contact also depend on water sources and its availability in the community.^28^

There was no significant association between proximity to the nearest water source, and schistosomiasis infection in the current study and this does not concur with previous studies which revealed that community proximity to an open water source showed a very strong association with infection. This could be attributed to the fact that majority of the respondents were living within the vicinity of the water bodies given that the area is majorly an agricultural area. In a study by^29^ which reported that fetching of water and living close to a stream and/or a water pool were identified as significant risk factors for Schistosomiasis infections. Studies show that human contact with *cercariae* infested water cause *Schistosoma* infection hence prevention of such water contact can prevent the transmission of the parasite. However, researchers argue that though safe water supplies reduce such water contact, total prevention of the parasite may not be possible due to the difference proportion of water contact geared by the different culture, socio economic differences and environmental factors.^26^

### Limitations

The study site being largely an irrigation scheme might show a high prevalence. We did not carry out a comparative study with a different study area that could have given us a better picture on the effects of environment and schistosomiasis infection. Participants might have altered their response or might have had a recall bias on whether they had suffered from schistosomiasis. The results should therefore be interpreted with caution.

## CONCLUSIONS/SIGNIFICANCE

Results from this study suggest that flood prevention and mitigation strategies need to be put in place in flood prone areas because these populations are exposed to greater health problems like communicable diseases e.g schistosomiasis. Providing efficient health education to people residing in schistosomiasis endemic areas is imperative for an effective and sustainable control programme. Our review suggests that increasing access to safe water and adequate sanitation are important measures to reduce the odds of schistosome infection.

The study recommends designing and construction of safe water sources in order to match the goal of effectively controlling and reversing the trends of schistosomiasis infections. The community should be made aware of the risk factors of schistosomiasis alongside raising health-seeking behaviours for diagnosis and treatment of schistosomiasis as a way of reducing the spread of infection.

## References

[1] Savioli, L., Daumiere, D., & Savioli, D. D. and L. (2012). Accelerating Work to Overcome the Global Impact of Neglected Tropical Diseases: A Roadmap for Implementation (World Health Organizaiton, Geneva, Switzerland, 2012). WHO. http://www.who.int/neglected_diseases/NTD_RoadMap_2012_Fullversion.pdf

[2] WHO. Investing to Overcome the Global Impact of Neglected Tropicaldiseases. Third WHO Report on Neglected Diseases. 2015;2015. 10.4324/9780429049194-1.

[3] French MD, Evans D, Fleming FM, et al. Schistosomiasis in Africa: Improving strategies for long-term and sustainable morbidity control. PLoS Negl Trop Dis. 2018;12(6):e0006484. 10.1371/journal.pntd.0006484. Medline 29953454 PMC6023105

[4] Sassa M, Chadeka EA, Cheruiyot NB, et al. Prevalence and risk factors of Schistosoma mansoni infection among children under two years of age in Mbita, Western Kenya. PLoS Negl Trop Dis. 2020;14(8):e0008473. 10.1371/journal.pntd.0008473. Medline 32841228 PMC7447014

[5] Chadeka EA, Nagi S, Sunahara T, et al. Spatial distribution and risk factors of Schistosoma haematobium and hookworm infections among schoolchildren in Kwale, Kenya. PLoS Negl Trop Dis. 2017;11(9):e0005872. 10.1371/journal.pntd.0005872. Medline 28863133 PMC5599053

[6] Masaku J, Madigu N, Okoyo C, Njenga SM. Current status of Schistosoma mansoni and the factors associated with infection two years following mass drug administration programme among primary school children in Mwea irrigation scheme: A cross-sectional study. BMC Public Health. 2015;15(1):739. 10.1186/s12889-015-1991-z. Medline 26231050 PMC4522152

[7] Okoyo C, Campbell SJ, Williams K, Simiyu E, Owaga C, Mwandawiro C. Prevalence, Intensity and Associated Risk Factors of Soil-Transmitted Helminth and Schistosome Infections in Kenya: Impact Assessment after Five Rounds of Mass Drug Administration in Kenya. Vol 14.; 2020. 10.1371/journal.pntd.0008604 PMC7540847 33027264

[8] Gichuki PM, Kepha S, Mulewa D, et al. Association between schistosoma mansoni infection and access to improved water and sanitation facilities in Mwea, Kirinyaga County, Kenya. BMC Infect Dis. 2019;19(1):503. 10.1186/s12879-019-4105-1. Medline 31174478 PMC6556037

[9] Kenya National Bureau of Statistics. 2019 Kenya Population and Housing Census Volume 1: Population by County and Sub-County. Vol I.; 2019. https://www.knbs.or.ke/?wpdmpro=2019-kenya-population-and-housing-census-volume-i-population-by-county-and-sub-county

[10] County Government of Kirinyaga. County Integrated Development Plan 2018-2022. Report. Published online 2018.

[11] KNBS S. Exploring Kenya's Inequality: Pulling Apart or Pooling Together? 2013. http://www.inequalities.sidint.net/Kenya/wp-content/uploads/sites/2/2013/09/Kirinyaga.pdf

[12] Fisher LD. Self-designing clinical trials. Stat Med. 1998;17(14):1551–1562. 10.1002/(SICI)1097-0258(19980730)17:14<1551::AID-SIM868>3.0.CO;2-. Medline 9699229

[13] Olson K, Stange M, Smyth J. Assessing within-household selection methods in household mail surveys. Public Opin Q. 2014;78(3):656–678. 10.1093/poq/nfu022.

[14] IBM Corp. Statistical Package for Social Scientists. 2015;23.

[15] Ng'ang'a M, Matendechero S, Kariuki L, et al. Spatial distribution and co-infection with urogenital and intestinal schistosomiasis among primary school children in Migori County, Kenya. East Afr Med J. 2016;93(10):S22–S31.

[16] Ismail HAHA, Hong ST, Babiker ATEB, et al. Prevalence, risk factors, and clinical manifestations of schistosomiasis among school children in the White Nile River basin, Sudan. Parasit Vectors. 2014;7(1):478. 10.1186/s13071-014-0478-6. Medline 25312470 PMC4200116

[17] Anto F, Asoala V, Adjuik M, Anyorigiya T, Oduro A, Akazili J. Water Contact Activities and Prevalence of Schistosomiasis Infection among School-age Children in Communities along an Irrigation Scheme in Rural Northern Ghana. J Bacteriol Parasitol. 2013;04(04). 10.4172/2155-9597.1000177.

[18] Agnew-Blais J, Carnevale J, Gropper A, Shilika E, Bail R, Ngoma M. Schistosomiasis haematobium prevalence and risk factors in a school-age population of peri-urban Lusaka, Zambia. J Trop Pediatr. 2010;56(4):247–253. 10.1093/tropej/fmp106. Medline 19892835

[19] Sady H, Al-Mekhlafi HM, Atroosh WM, et al. Knowledge, attitude, and practices towards schistosomiasis among rural population in Yemen. Parasit Vectors. 2015;8(1):436. 10.1186/s13071-015-1050-8. Medline 26302747 PMC4548916

[20] Salawu OT, Odaibo AB. Schistosomiasis transmission; sociodemographic, knowledge and practices as transmission risk factors in pregnant women. J Parasit Dis. 2016;40(1):93–99. 10.1007/s12639-014-0454-2. Medline 27065605 PMC4815848

[21] Kabatereine N, Fleming F, Thuo W, Tinkitina B, Tukahebwa EM, Fenwick A. Community perceptions, attitude, practices and treatment seeking behaviour for schistosomiasis in L. Victoria islands in Uganda. BMC Res Notes. 2014;7(1):900. 10.1186/1756-0500-7-900. Medline 25495121 PMC4307169

[22] Adoka SO, Anyona DN, Abuom PO, et al. East african MEdical Journal Prevalence And Control In Relation To Aquatic Habitats In The Lake Victoria Basin Of Kenya Community Perceptions Of Schistosomiasis Transmission, Prevalence And Control In Relation To Aquatic Habitats In The Lake Victoria Basi. Community Perceptions Schistosomiasis Transm. 2014;91(7). 26862658

[23] Liu L, Yang GJ, Zhu HR, Yang K, Ai L. Knowledge of, attitudes towards, and practice relating to schistosomiasis in two subtypes of a mountainous region of the People's Republic of China. Infect Dis Poverty. 2014;3(1):16. 10.1186/2049-9957-3-16. Medline 24955240 PMC4064289

[24] Yirenya-Tawiah, D. R., Annang, T., Otchere, J., Bentum, D., Edoh, D., Amoah, C., & Bosompem, K. M. (2011). Urinary schistosomiasis among adults in the Volta Basin of Ghana: prevalence, knowledge and practices. Journal of Tropical Medicine & Parasitology, 34(1), 1–16.

[25] Schrader M, Hauffe T, Zhang Z, et al. Spatially explicit modeling of schistosomiasis risk in eastern China based on a synthesis of epidemiological, environmental and intermediate host genetic data. PLoS Negl Trop Dis. 2013;7(7):e2327. 10.1371/journal.pntd.0002327. Medline 23936563 PMC3723594

[26] Grimes JET, Croll D, Harrison WE, Utzinger J, Freeman MC, Templeton MR. The roles of water, sanitation and hygiene in reducing schistosomiasis: a review. Parasit Vectors. 2015;8(1):156. 10.1186/s13071-015-0766-9. Medline 25884172 PMC4377019

[27] Kabuyaya M, Chimbari MJ, Manyangadze T, Mukaratirwa S. Schistosomiasis risk factors based on the infection status among school-going children in the Ndumo area, uMkhanyakude district, South Africa. S Afr J Infect Dis. 2017;32(2):67–72. 10.1080/23120053.2016.1266139.

[28] Manz KM, Kroidl I, Clowes P, et al. Schistosoma haematobium infection and environmental factors in Southwestern Tanzania: A cross-sectional, population-based study. PLoS Negl Trop Dis. 2020;14(8):e0008508. 10.1371/journal.pntd.0008508. Medline 32833959 PMC7446842

[29] Dawaki S, Al-Mekhlafi HM, Ithoi I, et al. Prevalence And Risk Factors Of Schistosomiasis Among Hausa Communities In Kano State, Nigeria. Rev Inst Med Trop São Paulo. 2016;58(0):54. 10.1590/S1678-9946201658054. Medline 27410914 PMC4964323

